# Identifying psychosocial predictors and developing a risk score for preterm birth among Kenyan pregnant women

**DOI:** 10.1186/s12884-024-07058-x

**Published:** 2025-01-02

**Authors:** Anna Larsen, Jillian Pintye, Felix Abuna, Julia C. Dettinger, Laurén Gomez, Mary M. Marwa, Nancy Ngumbau, Ben Odhiambo, Barbra A. Richardson, Salphine Watoyi, Joshua Stern, John Kinuthia, Grace John-Stewart

**Affiliations:** 1 Department of Epidemiology, University of Washington, 3980 15th Ave NE, Box 351619, Seattle, WA 98195 USA; 2 Department of Psychiatry and Behavioral Sciences, University of Washington, Seattle, WA USA; 3 Department of Global Health, University of Washington, Seattle, WA USA; 4 Department of Biobehavioral Nursing and Health Informatics, University of Washington, Seattle, WA USA; 5 University of Nairobi, Nairobi, Kenya; 6 Department of Research and Programs, Kenyatta National Hospital, Nairobi, Kenya; 7 Department of Biostatistics, University of Washington, Seattle, WA USA; 8 School of Medicine, Department of Pediatrics, University of Washington, Seattle, WA USA; 9 School of Medicine, Department of Allergy and Infectious Disease, University of Washington, Seattle, WA USA

**Keywords:** Risk score, Preterm birth, Kenya, Maternal depression, Birth outcomes

## Abstract

**Background:**

Preterm birth (PTB) is a leading cause of neonatal mortality, particularly in sub-Saharan Africa where 40% of global neonatal deaths occur. We identified and combined demographic, clinical, and psychosocial correlates of PTB among Kenyan women to develop a risk score.

**Methods:**

We used data from a prospective study enrolling HIV-negative women from 20 antenatal clinics in Western Kenya (NCT03070600). Depressive symptoms were assessed by study nurses using the Center for Epidemiologic Studies Depression Scale (CESD-10), intimate partner violence (IPV) with the Hurt, Insult, Threaten, Scream scale (HITS), and social support using the Medical Outcomes Survey scale (MOS-SSS). Predictors of PTB (birth < 37 weeks gestation) were identified using multivariable Cox proportional hazards models, clustered by facility. We used stratified k-fold cross-validation methods for risk score derivation and validation. Area under the receiver operating characteristic curve (AUROC) was used to evaluate discrimination of the risk score and Brier score for calibration.

**Results:**

Among 4084 women, 19% had PTB (incidence rate: 70.9 PTB per 100 fetus-years (f-yrs)). Predictors of PTB included being unmarried (HR:1.29, 95% CI:1.08–1.54), lower education (years) (HR:0.97, 95% CI:0.94–0.99), IPV (HITS score ≥ 5, HR:1.28, 95% CI:0.98–1.68), higher CESD-10 score (HR:1.02, 95% CI:0.99–1.04), lower social support score (HR:0.99, 95% CI:0.97–1.01), and mild-to-severe depressive symptoms (CESD-10 score ≥ 5, HR:1.46, 95% CI:1.07–1.99). The final risk score included being unmarried, social support score, IPV, and MSD. The risk score had modest discrimination between PTB and term deliveries (AUROC:0.56, 95% CI:0.54–0.58), and Brier Score was 0.4672. Women considered “high risk” for PTB (optimal risk score cut-point) had 40% higher risk of PTB (83.6 cases per 100 f-yrs) than “low risk” women (59.6 cases per 100 f-ys; HR:1.6, 95% CI:1.2–1.7, *p* < 0.001).

**Conclusion:**

A fifth of pregnancies were PTB in this large multi-site cohort; PTB was associated with several social factors amenable to intervention. Combining these factors in a risk score did not predict PTB, reflecting the multifactorial nature of PTB and need to include other unmeasured factors. However, our findings suggest PTB risk could be better understood by integrating mental health and support services into routine antenatal care.

**Supplementary Information:**

The online version contains supplementary material available at 10.1186/s12884-024-07058-x.

## Introduction

Preventable neonatal deaths account for nearly half of all deaths among children under five, totaling 2.4 million per year [1]. Many (43%) global neonatal deaths occur in in low- and middle-income countries (LMICs) of sub-Saharan Africa (SSA) [1], where gains in neonatal survival have been slowest [2, 3]. Preterm birth (PTB) contributes to neonatal deaths, particularly in low-resource settings of Africa where 40% of all neonatal deaths occurred among preterm births between 2012 and 2016 [4–6]. High attendance to maternal child health (MCH) care has improved maternal and child health survival across SSA, increasing the proportion of under 5 deaths attributable to the neonatal period [7]. New approaches to alleviating persisting disparities in neonatal morbidity and mortality are needed to achieve “last mile” gains in MCH.

Multiple factors are associated with increased risk of PTB, including maternal age (women < 18 and > 35 are at higher risk for PTB) [8], inadequate antenatal care attendance [9], and comorbidities (e.g., diabetes) [10]. Psychosocial factors such as depression [11], anxiety [12], and chronic stress [13, 14] increase risk of adverse perinatal outcomes, and risk may be further exacerbated by experiencing violence by an intimate partner (IPV) [15, 16]. Identifying risk factors for adverse perinatal outcomes can help prioritize pregnant women for enhanced monitoring in antenatal care, yet independent risk factors alone may be insufficient in screening for heightened risk when some factors may be additive.

Clinical prediction tools, such as “risk scores” or “calculators” are increasingly used in a range of healthcare settings to identify clients at increased risk of adverse health outcomes [17]. These tools offer a low-resource, efficient approach to aid healthcare workers in evaluating a client’s risk factors, assigning a score to differentiate “high” versus “low” overall risk for the outcome, and prioritizing “high risk” clients for enhanced monitoring and services. They may have utility in resource-limited settings to prioritize constrained healthcare worker time and services. While some risk factors for PTB are well documented, to date there is no risk scoring tool to identify pregnant women in SSA settings with high risk for PTB who may benefit from enhanced antenatal care. A few risk scores for PTB exist to date, yet these were predominantly developed in high-income settings, have low-to-moderate predictive capacity, and rely on factors less relevant in SSA settings such as smoking status [18–20]. One PTB prediction tool developed in Ethiopia included factors measured at the time of delivery, making it less useful for early detection and monitoring [21]. The persisting high risk of PTB in Kenya combined with high antenatal care attendance (> 90%) [22], make Kenya an ideal setting for development and validation of a risk scoring tool to predict PTB.

We aimed to derive and validate a novel clinical tool using a combination of maternal factors feasibly obtained within routine maternal child health care to identify pregnancies at higher risk for PTB among pregnant women in Western Kenya.

## Methods

### Study design and participants

This study was nested this analysis in the PrEP Implementation for Mothers in Antenatal Care study (PrIMA) conducted among pregnant women in Western Kenya to compare two models for pre-exposure prophylaxis implementation in a cluster randomized controlled trial (NCT03070600) [23]. We screened and enrolled women attending antenatal care (ANC) in 20 MCH clinics in Siaya and Homa Bay counties between January 15, 2018, and July 31, 2019. Women were eligible for the PrIMA study if they were pregnant, HIV-uninfected, ≥ 15 years old and were able to provide consent.

### Study procedures

Data about demographics, pregnancy history, partner characteristics, and psychosocial factors were collected by study nurses through questionnaires administered in Kiswahili, Dholuo, or English languages with tablet-based REDCap surveys. Gestational age at enrollment was assessed by study nurses via ascertainment of last menstrual period (LMP) or fundal height. Study nurses collected data on pregnancy outcomes at the 6-week postpartum visit and abstracted data from medical records at study visits that aligned with antenatal and infant immunization visits recommended by the Kenya Ministry of Health. Visits occurred monthly during pregnancy, at 6 weeks postpartum, and three-monthly until 9 months postpartum.

### Outcomes

The primary outcome was PTB, defined as a live or non-live birth < 37 weeks gestation. Gestational age was determined by study nurses at enrolment by ascertainment of last menstrual period (LMP) or fundal height (which was assessed in 2% of participants). In study settings where ultrasound is typically unavailable, LMP is used as a standard [24]. 

### Potential predictors

We hypothesized multiple demographic, clinical, and social characteristics as potential predictors of adverse perinatal outcomes, including PTB, based on existing evidence (Supplementary Material Fig. 1). The Center for Epidemiologic Studies Depression Scale (CESD-10) was used to collect information about experience of depressive symptoms during pregnancy (enrollment visit). In this 10-item scale, higher severity of depressive symptoms is denoted by higher scores (absolute score range: 0–30) and a cut-point of 10 or greater denotes moderate-to-severe depressive symptoms (MSD, CESD-10 score ≥ 10) [25]. To identify differences between women with “any” versus “no” depressive symptoms, we evaluated mild-to-severe depressive symptoms (Mild-SD), defined as a cut-point of 5 or greater (CESD-10 score ≥ 5). The 18-item Medical Outcomes Study Social Support Survey (MOS-SSS) was used to collect social support data; higher scores describe higher social support (range: 18–90) [26]. To determine history of intimate partner violence (IPV), we used the 4-item Hurt, Insult, Threaten, Scream Scale with a cut-point of 10 or greater (absolute score range: 4–20) [27]. 

Experience of prior PTB was self-reported, as was diagnosis of sexually transmitted infection (prior 6 months). Syphilis diagnosis information was abstracted from the patient’s medical card at enrollment or serologically evaluated. Household crowding was used as a marker of socioeconomic status [28], defined as the ratio of people per room in a residence greater than the median (> 2 people/room). Maternal comorbidity was defined as self-report of diabetes, hypertension, or tuberculosis.

Overall, we evaluated the following potential predictors of PTB: Maternal age (years, continuous), educational attainment (years, continuous), regular employment (yes/no), being married or living with a partner (yes/no), household crowding (yes/no), financial support from partner (yes/no), maternal comorbidity (yes/no), recent STI (yes/no), recent syphilis infection (yes/no), multiparity (yes/no), prior preterm birth, IPV in last 2 weeks (yes/no), social support score (continuous), moderate-to-severe depressive symptoms (yes/no), mild-to-severe depressive symptoms (yes/no), depressive symptom score (continuous).

### Statistical analysis -- Risk score derivation and validation

Participants were included in this analysis if they had a singleton birth, had information on gestational age at birth (implausible values of gestational age > 44 weeks were considered missing), did not acquire HIV during the study, and had depressive symptom information during pregnancy.

We used stratified k-fold cross-validation methods for risk score derivation and validation [29–32]. Cox proportional hazards models clustered by facility were used to identify potential predictors of PTB (*p* < 0.10), which were included in a multivariable model. A stepwise backwards Cox proportional hazards regression approach was used to identify the combination of variables that best predicted PTB, starting with the variables included in the multivariable model. Dichotomized versions of continuous variables were evaluated by identifying the optimal cut-point through signal detection receiver operating characteristic curve (ROC) analysis (Stata “cutpt” command) [33]. We used dichotomized versions if they were more predictive than the continuous version. This was the case for HITS score, where the most predictive cut-point was HITS score ≥ 5 versus < 5.

All variables were defined so that the direction of their association was a “risk factor” for PTB, except for continuous variables where higher scores were associated with lower risk of PTB (these variables had negative item-level scores). The model with lowest Akaike information criterion (AIC) from the models evaluated in stepwise analysis was identified as the best fit model for the risk score [34]. To calculate a score value for each predictor from the best fit model, we divided each factor’s regression coefficient by the absolute value of the lowest value coefficient, then rounded to the nearest integer. The final risk score for each woman was the sum of individual predictor scores.

To understand the extent to which the risk score differentiated women experiencing PTB from women without this experience (discrimination), we used ROC curve analysis to calculate area under the receiver operating characteristic curve (AUROC) for the risk score as a predictor of PTB [17, 32]. To understand how closely the predictions of the risk score match the observed outcomes in the data (calibration), we calculated a Brier score [17, 32]. Overall, useful risk scores have AUROC > 0.70 and Brier score < 0.25.

To internally validate the risk score, we first split the data into 10 equally sized random subsets (“folds”) that were stratified by PTB to ensure equal distribution of outcome risk in each fold. Meaningful differences were not found between participants in each fold. In 10 iterations using datasets comprised of 9 out of 10 folds each (90% of full dataset), we repeated all model derivation steps from the main risk score development process. Each of the 10 resulting risk scores were tested for their performance predicting PTB in the fold not used to derive that risk score (10% of full dataset). Estimated AUROC, sensitivity, specificity (at their respective optimal cut-points), and Brier score for each risk score were averaged to obtain overall measures of model performance in internal validation to which we compared the main risk score performance characteristics. Risk scores were dichotomized into the most predictive categories using the optimal cut-point identified through ROC curves and evaluated incidence of PTB by risk score group.

We imputed item-level scores for participants missing data in < 5 out of 10 depressive symptom scale items (11.8%, 492/4185, Supplementary Material Table 1) using the median score across the participant’s existing items (person-median imputation) [35]. We imputed item-level scores using person-median imputation among those missing < 8 out of 16 social support scale items (2.6%, 108/4185, Supplementary Material Table 2).

### Sensitivity analyses

In sensitivity analyses, we evaluated the risk score among sub-groups stratified by trimester of enrollment to understand if the risk score was more predictive of PTB in particular trimesters. We additionally evaluated the risk score among groups stratified by adolescent and young adult women (< 25 years) versus adult women (≥ 25 years). Analyses were conducted using Stata 15.

### Ethical considerations

We received approval from the Kenyatta National Hospital-University of Nairobi Ethics Research Committee and University of Washington Human Subjects Review Committee for the study protocol, informed consent forms, and data collection tools. The study received approval from Siaya and Homabay county Departments of Health and facility administrators. All participants provided written informed consent.

## Results

Overall, 4084 (92%, 4084/4447) PrIMA study participants were included in this analysis. These participants had information about PTB outcome, did not acquire HIV during the study, had a singleton birth, and had depressive symptom information during pregnancy. Median maternal age was 24 years (interquartile range [IQR]: 21–28), median gestational age at enrollment in PrIMA was 24 weeks (IQR: 20–29), and median educational attainment was 10 years (IQR: 8–12) (Table 1). The majority of participants were multiparous (74.4%, 3035/4084) and married or living with their partner (84.8%, 3433/4084).

**Table 1:** Baseline characteristics of PrIMA study participants included in analysis

	Overall (*n* = 4084)		Preterm birth (*n* = 780)		No preterm birth (*n* = 3304)	
**Demographic characteristics**	**N**	*n* (%) or Median (IQR)	*n*	*n* (%) or Median (IQR)	*n*	*n* (%) or Median (IQR)
Age (years)	4082	24 (21, 28)	780	24 (21, 28)	3302	24 (21, 28)
Adolescents and young women (≤ 24 years)	4082	2332 (57.1%)	780	467 (59.9)	3302	1865 (56.5)
Gestational age (enrollment, weeks)	4084	24 (20, 29)	780	24 (18, 28)	3304	24 (20, 30)
Married or living with a partner	4050	3433 (84.8%)	777	652 (83.9)	3273	2781 (85.0)
Completed education (years)	4084	10 (8, 12)	768	10 (8, 12)	3235	10 (8, 12)
Regularly employed	4032	603 (15.0%)	772	118 (15.3)	3260	485 (14.9)
Financial support from partner	4084	3616 (88.5%)	780	694 (89.0)	3304	2922 (88.4)
Household crowding (≥ 2 people/room)	4057	1960 (48.3%)	777	378 (48.6)	3280	1582 (48.2)
**Pregnancy history & reproductive health factors**						
Multiparous	4079	3035 (74.4%)	779	581 (74.6)	3300	2454 (74.4)
Prior preterm birth	4084	42 (1.0%)	780	6 (0.8%)	3304	36 (1.1)
Maternal comorbidity	4084	45 (1.1%)	780	11 (1.4)	3304	34 (1.0)
Trimester of initial antenatal care (ANC) visit	4084		780		3304	
First		610 (14.9%)		121 (15.5)		489 (14.8)
Second		2092 (51.2%)		443 (56.8)		1649 (49.9)
Third		1382 (33.8%)		216 (27.7)		1166 (35.3)
Sexually transmitted infection (prior 6 months)	4077	103 (2.5%)	779	16 (2.1)	3298	87 (2.6)
Syphilis infection (enrollment)	4016	42 (1.0%)	757	8 (1.1)	3259	34 (1.0)
**Psychosocial characteristics**						
Social support score^b^	4084	75 (63, 88)	780	73 (58.5, 86)	3304	76 (64, 88)
Intimate partner violence ^c^ (HITS score ≥ 10)	4079	318 (7.8%)	778	48 (6.2)	3301	270 (8.2)
CESD-10 score	4084	5 (3, 9)	780	6 (3, 10)	3304	773 (23.4)
Mild-to-severe depressive symptoms (CESD-10 ≥ 5)	4084	2239 (54.8%)	780	492 (63.1)	3304	1747 (52.9)
Moderate-to-severe depressive symptoms (CESD-10 ≥ 10)	4084	975 (23.9%)	780	202 (25.9)	3304	5 (2, 9)

*Among those with current partners

^b^We evaluated social support using the 18-item Medical Outcomes Study social support score (MOS-SSS)

^c^We evaluated intimate partner violence using the 4-item Hurt, Insult, Threaten, and Scream scale (HITS), defining intimate partner violence as scores of 10 and above (IPV: HITS score ≥ 10 = “Yes”, HITS score < 10 = “No”)

CESD-10: Center for epidemiologic studies depression scale-10

MSD: Moderate-to-severe depressive symptoms

About a quarter of women (23.9%, 975/4084) reported MSD during pregnancy (median CESD-10 score: 5, IQR: 3–9), and over 50% of women reported mild-SD (54.8%, 2239/4084). Median MOS-SSS score was 75, IQR: 63–88), and 8% reported IPV at enrollment (7.8%, 318/4084). Prior to imputing CESD-10 and MOS-SSS items for partial scores (missing < 50% of items) 36.9% of women had low social support (median social support score: 75, IQR: 63–88) and 24.6% of women had MSD (median CESD-10 score: 5, IQR: 3–9). Those with complete vs. partial CESD-10 data were not meaningfully different in most factors evaluated (Supplementary Material Table 3).

### Risk score to predict preterm birth

Overall, one in five (19%, 780/4084) women experienced PTB over 1099.3 person-years of follow-up for an incidence rate of 70.9 cases per 100 fetus-years (95% Confidence Interval [CI]: 66.1–76.1). Risk of PTB was higher among women who were unmarried and not living with a partner (HR: 1.3, 95% CI: 1.1–1.6, *p* = 0.0035) compared to married women. On average, every additional year of educational attainment was associated with lower risk of PTB (HR: 0.97, 95% CI: 0.94–0.99, *p* = 0.0383). Women experiencing IPV (HITS score ≥ 5) had higher risk of PTB (HR: 1.3, 95% CI: 0.9–1.7, 0.0697) compared to those with lower HITS scores (< 5). Women with higher CESD-10 scores were at higher risk of PTB (HR: 1.02, 95% CI: 0.98–1.68, *p* = 0.0632), as well as those with mild-to-severe depressive symptoms (HR: 1.5, 95% CI: 1.1–1.9, *p* = 0.0157), compared to those reporting low or no depressive symptoms. Those with low social support (MOS-SSS score ≤ 75; identified by Stata “cutpt” command) had higher risk of PTB (HR: 1.3, 95% CI: 0.90–1.84, *p* = 0.1625) (Table 2). Being unmarried, experiencing IPV, having low social support, and having mild-to-severe depressive symptoms comprised those included in the model with lowest AIC in stepwise backwards Cox regression (Table 2).

**Table 2:** Correlates of preterm birth and item-level risk scores to predict preterm birth

Baseline characteristics	Univariate analysis		Multivariate analysis		Stepwise		Coefficient	Risk score
	Crude HR (95% CI)	*p*-value	Adjusted HR (95% CI)	*p*-value	Adjusted HR (95% CI)	*p*-value	β	
**Demographic characteristics**								
Age (years)	0.99 (0.97–1.01)	0.2526						
Adolescents and young women (< 24 years)	1.11 (0.92–1.35)	0.2740						
Not married or living with a partner	1.29 (1.08–1.54)	0.0044	1.34 (1.11–1.62)	0.0023	1.34 (1.12–1.62)	0.0018	0.29	2
Completed education (years)	0.97 (0.94–0.99)	0.0386						
Educational attainment ≥ 2.5 years	0.87 (0.74–1.03)	0.1087	0.99 (0.84–1.18)	0.9432				
Regularly employed	1.00 (0.65–1.56)	0.9901						
Financial support from partner	0.88 (0.67–1.14)	0.3366						
Household crowding (≥ 2 people/room)	1.01 (0.97–1.06)	0.5832						
**Pregnancy history & reproductive health factors**								
Multiparous	0.97 (0.79–1.20)	0.8003						
Prior preterm birth	0.64 (0.34–1.20)	0.1653						
Maternal comorbidity	1.24 (0.79–1.94)	0.3411						
Sexually transmitted infection (prior 6 months)	0.87 (0.49–1.54)	0.6289						
Syphilis infection (enrollment)	1.08 (0.50–2.29)	0.8511						
**Psychosocial characteristics**								
Social support score^a^	0.99 (0.97-1.00)	0.0953						
Low social support (MOS-SSS ≤ 75)^b^	1.29 (0.90–1.84)	0.1625	1.19 (0.82–1.72)	0.3629	1.19 (0.82–1.72)	0.3641	0.17	1
Intimate partner violence (HITS score ≥ 5)^c^	1.28 (0.98–1.68)	0.0697	1.22 (0.88–1.69)	0.2392	1.22 (0.88–1.69)	0.2400	0.19	1
CESD-10 score^d^	1.02 (0.99–1.04)	0.0632						
Mild-to-severe depressive symptoms (CESD-10 ≥ 5)^e^	1.46 (1.07–1.99)	0.0157	1.35 (0.98–1.87)	0.0650	1.35 (0.98–1.87)	0.0657	0.30	
Moderate-to-severe depressive symptoms (CESD-10 ≥ 10)^f^	1.14 (0.82–1.60)	0.4123						2

^a^We evaluated social support using the 18-item Medical Outcomes Study social support score (MOS-SSS)

^b^We evaluated low social support as MOS-SSS score ≤ 75 based on the Stata cutpt function which identified the most predictive cut-point for a dichotomized variable

^c^We evaluated intimate partner violence using the 4-item Hurt, Insult, Threaten, and Scream scale (HITS). In risk score development, we used a cutpoint of HITS score ≥ 5 vs. HITS score < 5 based on the most predictive cutpoint for preterm birth

^d^CESD-10: Center for epidemiologic studies depression scale-10

^e^Mild-to-severe depressive symptoms was defined as CESD-10 ≥ 5 vs. CESD-10 < 5

^f^MSD: Moderate-to-severe depressive symptoms was defined as CESD-10 ≥ 10 vs. CESD-10 < 10

**Fig. 1:**
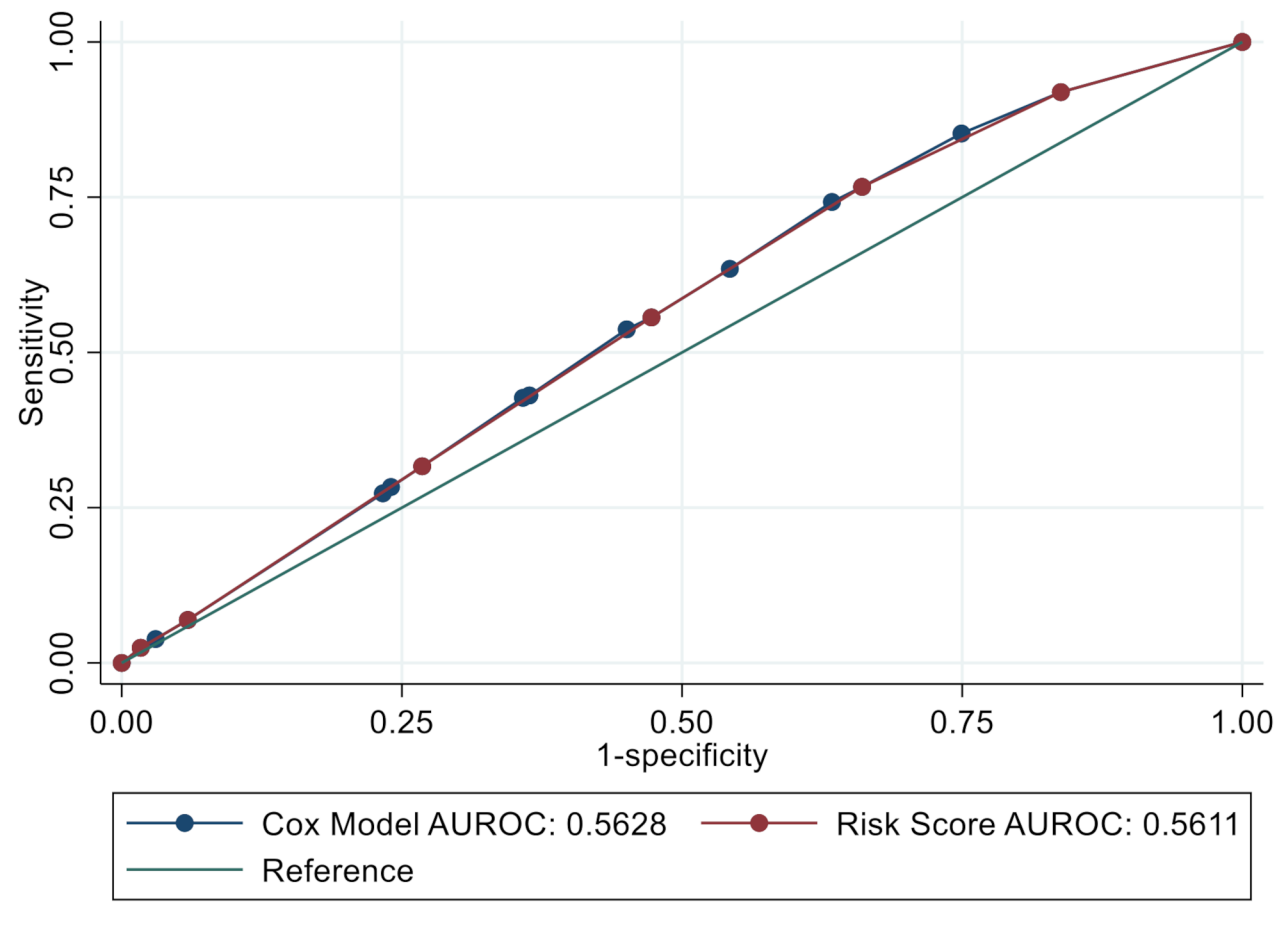
Receiver operating characteristic curve and cut-points of risk score

The ROC curves for the risk score and the multivariable model overlapped (Fig. 1), indicating equal predictive capacity. Median risk score among all women was 2 (IQR: 1–4, range: 0–6). The risk score had an AUROC of 0.56 (95% CI: 0.54–0.58). At the optimal cut-point (> 2.5 vs. ≤2.5), sensitivity was 55.6% (95% CI: 52.1–59.2) and specificity was 52.7% (95% CI: 51.0-54.4). The Brier score was 0.4672. Incidence of PTB differed between groups dichotomized by the optimal cut-point for the risk score, where incidence of PTB was 83.6 cases per 100 fetus-years among those with risk scores > 2.5, and 59.6 cases per 100 fetus-years among those with score ≤ 2.5 (HR: 1.4, 95% CI: 1.2–1.7, p-value < 0.001) (Table 3).

**Table 3:** Area under the receiver operating curve overall and by trimester of enrollment

	*N*	AUROC	95% CI
Overall	4084	0.561	0.540–0.582
1^st^ Trimester	406	0.573	0.507–0.639
2^nd^ Trimester	2059	0.551	0.523–0.579
3^rd^ Trimester	1619	0.579	0.544–0.614
Adolescents and young adults (< 25 years)	2332	0.547	0.519–0.574
Adult women (≥ 25 years)	1750	0.579	0.547–0.612

In sensitivity analyses, the discrimination of the risk score did not improve among groups defined by trimester of enrollment or in groups stratified by age group (< 25 years versus ≥ 25 years) (all AUCs < 0.58; Table 3).

### Risk score validation

After dividing the dataset into 10 random and equal subsets, we did not detect meaningful differences in characteristics between women included in these validation subsets or the overall dataset, and stratification ensured incidence of PTB was equal across groups (Table 4). The AUROC, sensitivities and specificities (at their respective optimal cut-points), and Brier scores were similar across validation subsets and the overall dataset. The mean AUROC across iterations was 0.56 (95% CI: 0.49–0.63) and mean Brier score was 0.5765. In all risk scores from validation subsets, the incidence of PTB in the group with “high risk” scores were statistically significantly higher than the incidence of PTB in the group with “low risk” scores (data not shown). Averaged across validation subsets, the sensitivity at the optimal risk score cut-point was 61.5% and specificity was 49.7%. The risk score performance characteristics were similar for the main risk score and the average metrics across validation subsets, indicating strong internal validity (Table 3).

**Table 4:** Discrimination Performance of Risk Score in Development and K-fold Cross-Validation cohorts

			Risk of preterm birth			Discrimination performance			
**Cohort**	*N*	**Women with high risk score** *n* (%)	No. of cases	Fetus-years	Incidence rate per 100 fetus-years (95% CI)	AUC (95% CI)	Brier score	Sensitivity*	Specificity*
**Overall (derivation)**	4084	2029 (48.9%)	780	1099.3	70.9 (66.1–76.1)	0.56 (0.54–0.58)	0.4672	55.6%	52.7%
**1**	408	**224 (53.9%)**	78	114.2	68.3 (54.7–85.2)	0.58 (0.56–0.60)	0.4730	67.9%	49.1%
**2**	408	**234 (56.4%)**	78	111.7	69.8 (55.9–87.1)	0.58 (0.55–0.59)	0.4755	71.8%	47.9%
**3**	408	**238 (78.0%)**	78	107.7	72.4 (58.0-90.4)	0.57 (0.55–0.60)	0.6545	85.5%	23.2%
**4**	409	**204 (49.6%)**	78	113.2	68.9 (55.2–86.0)	0.59 (0.56–0.61)	0.4938	50.0%	50.8%
**5**	409	**207 (49.8%)**	78	110.2	70.8 (56.7–88.4)	0.58 (0.56–0.60)	0.4719	57.7%	51.7%
**6**	408	**223 (53.7%)**	78	110.7	70.4 (56.4–87.9)	0.58 (0.56–0.60)	0.4975	59.0%	48.2%
**7**	408	**206 (49.6%)**	78	105.7	73.8 (59.1–92.1)	0.57 (0.55–0.59)	0.4436	65.4%	53.3%
**8**	408	**216 (52.1%)**	78	108.4	71.9 (57.6–89.9)	0.57 (0.55–0.60)	0.4730	62.8%	50.3%
**9**	409	**225 (54.1%)**	78	108.3	72.0 (57.7–89.9)	0.58 (0.56–0.60)	0.5061	59.0%	47.1%
**10**	409	**201 (49.1%)**	78	109.2	71.4 (57.2–89.2)	0.58 (0.56–0.60)	0.7818	55.2%	54.6%
**Average (validation)**	4084	**--**	--	--	--	0.56 (0.49–0.63)	0.5765	61.5%	49.7%

*Sensitivity and specificity at the optimal cut-point of each respective risk score

## Discussion

In this large, multi-site, longitudinal study among Kenyan pregnant women, we determined risk factors for PTB and endeavored to develop and validate a risk score to identify women at higher risk of PTB. We envisioned that this tool could be implemented in routine antenatal care settings in sub-Saharan Africa to prioritize women for enhanced care throughout pregnancy with the aim of preventing PTB and ultimately improving neonatal survival. Overall, 19% of births were preterm -- higher than PTB estimates for sub-Saharan Africa as a whole (12%) [36, 37], but similar to other studies in Kenya [38]. Several pregnancy and psychosocial predictors of PTB were identified, including being unmarried, having lower educational attainment, experiencing IPV, having lower social support, and experiencing mild-to-severe depressive symptoms. Despite these associations, the combination risk score was not useful in discriminating between cases of PTB and non-cases (AUC:0.57; minimum “useful” AUC: ≥0.70). Our findings contribute new information to inform future risk prediction tool development for this important population, with the goal of preventing PTB to improve neonatal survival.

A few risk assessment tools for adverse perinatal outcomes have been developed globally with low-to-moderate prediction accuracy [18, 19, 21, 39]. Most tools were developed in US or Europe and have limited utility in sub-Saharan African antenatal care clinics since they include clinical factors not routinely assessed in these settings, such as fetal fibronectin in vaginal fluid [19], or behavioral factors less common in SSA settings like smoking [20]. One risk score developed among Ethiopian pregnant women to predict PTB performed well (AUROC: 0.816) and included some maternal factors readily collected in routine ANC (residence, gravidity) as well as others less-routinely collected in public sector ANC (hemoglobin < 11 mg/dL, early rupture of membranes, antepartum hemorrhage, pregnancy-induced hypertension) [21]. Some of the variables included in this risk score were obtained at delivery, which limits utility for predicting women at-risk for PTB for care during the antenatal period [21]. These variables often occur simultaneously with PTB and may have the same pathophysiology which make them less useful for predicting PTB. We attempted to develop a model for use in earlier antenatal care to identify women earlier in pregnancy who may be at risk for PTB, using factors easily collected by lower cadre healthcare workers in routine sub-Saharan African antenatal care settings.

The predictors of PTB in our analysis were consistent with factors identified in prior studies. Unmarried women are at increased risk globally for preterm, low birthweight, and small-for-gestational age births [40, 41]. Linkages between lower educational attainment and heightened risk for PTB are well-documented, particularly in high-income country settings [42, 43], however a recent analysis of Demographic and Health Surveys from > 170,000 reproductive-aged women from 36 sub-Saharan African countries found that women with higher educational achievement had higher risk for PTB [41]. Our data support lower educational attainment as a risk factor for PTB. Increasingly, psychosocial factors including social support, depressive symptoms, and intimate partner violence are understood as important factors associated with adverse perinatal outcomes like PTB [11, 16, 44]. Our study supported these factors as potential risk factors for PTB among Kenyan pregnant women. Our study did not identify wealth index, employment status, prior PTB, or parity/gravidity as correlates of PTB.

Overall, while our study identified predictors of PTB that are routinely and/or easily collected in ANC clinics in SSA, the effect sizes in combination were not strong enough to discriminate cases from non-cases in our risk score. Regardless of the poor predictive performance, women with risk scores indicating “high risk” still had 60% higher risk of PTB compared to women with lower scores. Unmarried women with lower educational attainment and those experience intimate partner violence, depressive symptoms, or low social support be prioritized for enhanced monitoring throughout antenatal care. Routine screening for intimate partner violence and depressive symptoms should be integrated into maternal child health in sub-Saharan Africa as an important component of addressing maternal psychosocial distress to reduce risk of PTB.

### Limitations

We hypothesized that the poor performance of our risk score could be due to failure to account for selection bias in estimates of PTB, based on differential enrollment by exposure and outcome status among those enrolling later versus earlier in gestation. Therefore, a sensitivity analysis was conducted to evaluate discrimination of the risk score among groups defined by trimester of enrollment. This did not improve discrimination of the risk score (AUC for all trimesters: ≤0.59). Further, we hypothesized that the risk score may discriminate differentially among adolescent or young adult women (< 25) years compared to adult women (≥ 25 years), yet stratification by age did not improve discrimination (AUC for both age groups ≤ 0.59). Our risk score could have been improved by including other predictors that were not measured in our study, such as bleeding in pregnancy, hypertension, or indoor air quality [45]. 

## Conclusion

Among a large cohort of pregnant Kenyan women, a fifth of pregnancies were preterm, and this outcome was higher among women who were unmarried, those with lower educational attainment, and those with mild-to-severe depressive symptoms. Our risk score that combined marital status, years of education, and psychosocial factors of intimate partner violence, social support, and depressive symptoms did not yield high discriminatory capacity for predicting preterm birth. However, our data suggest subgroups of women who may benefit from increased antenatal support (unmarried, lower social support, IPV), and that integrating depression screening into antenatal care with appropriate referral for counseling or psychotherapy could potentially reduce risk for preterm birth.

## Electronic Supplementary Material

Below is the link to the electronic supplementary material.

Supplementary Material 1

Supplementary Material 2

## Data Availability

The datasets used during the current study are available from the corresponding author on reasonable request.
