# Supplementary material for: Identifying psychosocial predictors and developing a risk score for preterm birth among Kenyan pregnant women
Source: BMC Pregnancy Childbirth. 2025 Jan 2;25:2. doi: 10.1186/s12884-024-07058-x (PMC11697889; doi:10.1186/s12884-024-07058-x)
Supplement: Supplementary file 1 — Supplementary Material 1 [file 12884_2024_7058_MOESM1_ESM.docx]

Supplementary Material

**Figure 1. Conceptual model for the relationship between depression and adverse perinatal outcomes**


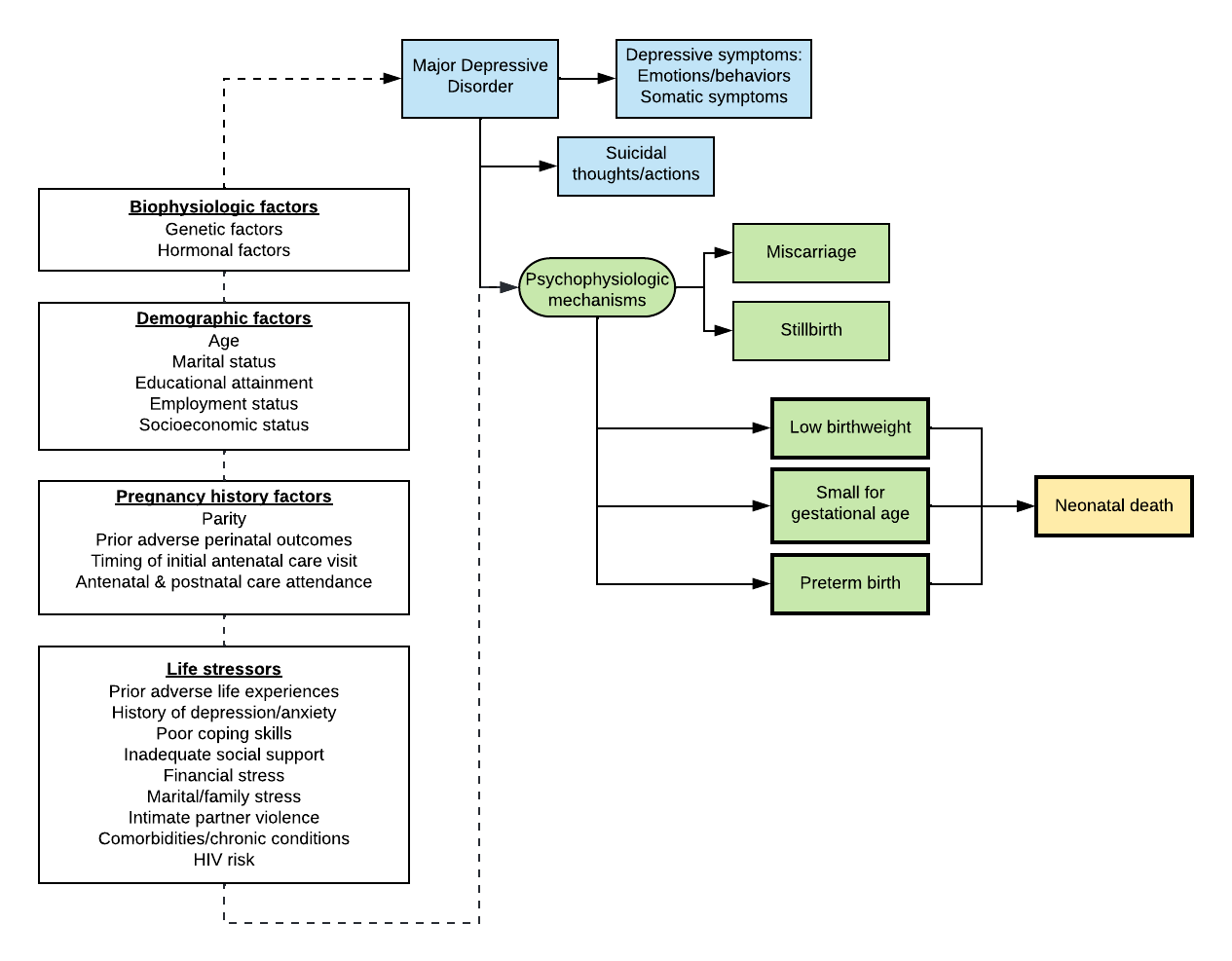


**Table 1. Number of CESD-10 items missing among singleton births with pregnancy outcome data among women who did not acquire HIV by 9 months postpartum (n=4185)**

| Number of CESD-10 items missing | N (%) |
| --- | --- |
| 0 | 3661 (87.48%) |
| 1 | 259 (6.19%) |
| 2 | 152 (3.63%) |
| 3 | 67 (1.60%) |
| 4 | 14 (0.33%) |
| 5 | 7 (0.17%) |
| 6 | 2 (0.05%) |
| 7 | 1 (0.02%) |
| 8 | 1 (0.02%) |
| 9 | 0 (0.00%) |
| 10 | 21 (0.50%) |

**Table 2. Number of MOS-SSS items missing among singleton births with pregnancy outcome data among women who did not acquire HIV by 9 months postpartum (n=4185)**

| Number of MOS-SSS items missing | N (%) |
| --- | --- |
| 0 | 4077 (97.42%) |
| 1 | 80 (1.91%) |
| 2 | 7 (0.17%) |
| 3 | 3 (0.07%) |
| 4 | 18 (0.43%) |

**Table 3. Baseline characteristics of PrIMA study participants among those with complete CESD-10 information versus partial (n=4153)**

|  | **Overall**  (N=4153) | **Complete CESD-10** (N=3659) | **Incomplete CESD-10**  (N=492) |  |
| --- | --- | --- | --- | --- |
| **Demographic characteristics** | n (%) or  Median (IQR) | n (%) or Median (IQR) | n (%) or Median (IQR) |  |
| Age (years) | 24 (21, 28) (n=4151) | 24 (21, 29) (n=3659) | 24 (21, 28) (n=492) | 0.23 |
| Adolescents and young women (<24 years) | 2370 (57.1%) | 2081 (56.8%) | 289 (58.7%) | 0.43 |
| Missing | 2 (<1%) | 2 (0.1%) | 0 (0.0%) |  |
| Gestational age (enrollment, weeks) | 24 (20, 30) (n=4153) | 24 (20, 30) (n=3661) | 26 (18.285714, 30) (n=492) | 0.28 |
| Married or living with a partner | 3491 (84.1%) | 3083 (84.2%) | 408 (82.9%) | 0.27 |
| Missing | 34 (0.8%) | 33 (0.9%) | 1 (0.2%) |  |
| Completed education (years) | 10 (8, 12) (n=4072) | 10 (8, 12) (n=3589) | 10 (8, 12) (n=483) | 0.34 |
| Regularly employed | 612 (14.7%) | 517 (14.1%) | 95 (19.3%) | 0.002 |
| Missing | 52 (1.3%) | 44 (1.2%) | 8 (1.6%) |  |
| Household crowding (≥2 people/room) | 1995 (48.0%) | 1771 (48.4%) | 224 (45.5%) | 0.20 |
| Missing | 27 (0.7%) | 26 (0.7%) | 1 (0.2%) |  |
| **Pregnancy history & factors** |  |  |  |  |
| Multiparous | 3082 (74.2%) | 2735 (74.7%) | 348 (70.7%) | 0.062 |
| Missing | 5 (0.1%) | 4 (0.1%) | 1 (0.2%) |  |
| Prior pregnancy loss | 539 (13.0%) | 468 (12.8%) | 71 (14.4%) | 0.30 |
| Missing | 14 (0.3%) | 12 (0.3%) | 2 (0.4%) |  |
| Prior preterm birth | 42 (1.0%) | 35 (1.0%) | 7 (1.4%) | 0.33 |
| Trimester of initial antenatal care (ANC) visit |  |  |  |  |
| First | 615 (14.8%) | 523 (14.3%) | 92 (18.7%) | <0.001 |
| Second | 2098 (50.5%) | 1894 (51.7%) | 204 (41.5%) |  |
| Third | 1440 (34.7%) | 1244 (34.0%) | 196 (39.8%) |  |
| Infant sex (female) | 1841 (44.3%) | 1632 (44.6%) | 209 (42.5%) | 0.62 |
| Missing | 535 (12.9%) | 463 (12.6%) | 72 (14.6%) |  |
| **HIV risk factors** |  |  |  |  |
| Self-perceived high HIV risk | 369 (8.9%) | 320 (8.7%) | 49 (10.0%) | 0.35 |
| Missing | 7 (0.2%) | 4 (0.1%) | 3 (0.6%) |  |
| Lifetime sexual partners | 2 (2, 3) (n=4148) | 2 (2, 3) (n=3656) | 2 (2, 3) (n=492) | 0.59 |
| Partner HIV-positive* | 176 (4.2%) | 149 (4.1%) | 27 (5.5%) | 0.15 |
| Missing | 52 (1.3%) | 49 (1.3%) | 3 (0.6%) |  |
| Sexually transmitted infection (enrollment) | 104 (2.5%) | 96 (2.6%) | 8 (1.6%) | 0.19 |
| Missing | 7 (0.2%) | 5 (0.1%) | 2 (0.4%) |  |
| **Psychosocial characteristics** |  |  |  |  |
| Low social support (MOS-SSS score <72) | 1504 (36.2%) | 1372 (37.5%) | 132 (26.8%) | <0.001 |
| Missing | 90 (2.2%) | 69 (1.9%) | 21 (4.3%) |  |
| Intimate partner violence ^c^ (HITS score ≥10) | 323 (7.8%) | 289 (7.9%) | 34 (6.9%) | 0.44 |
| Missing | 5 (0.1%) | 5 (0.1%) | 0 (0.0%) |  |
